# Particle Morphology of Medusavirus Inside and Outside the Cells Reveals a New Maturation Process of Giant Viruses

**DOI:** 10.1128/jvi.01853-21

**Published:** 2022-03-17

**Authors:** Ryoto Watanabe, Chihong Song, Yoko Kayama, Masaharu Takemura, Kazuyoshi Murata

**Affiliations:** a School of Life Science, The Graduate University for Advanced Studies, Okazaki, Aichi, Japan; b Exploratory Research Center on Life and Living Systems, National Institutes of Natural Sciences, Okazaki, Aichi, Japan; c National Institute for Physiological Sciences, National Institutes of Natural Sciences, Okazaki, Aichi, Japan; d Terabase, Inc., Okazaki, Aichi, Japan; e Institute of Arts and Sciences, Tokyo University of Science, Shinjuku, Tokyo, Japan; University of Illinois at Urbana-Champaign

**Keywords:** cryo-electron microscopy, giant virus, NCLDV, single-particle analysis, electron tomography, amoeba cell, virus maturation

## Abstract

Medusavirus, a giant virus, is phylogenetically closer to eukaryotes than the other giant viruses and has been recently classified as an independent species. However, details of its morphology and maturation process in host cells remain unclear. Here, we investigated the particle morphology of medusavirus inside and outside infected cells using conventional transmission electron microscopy (C-TEM) and cryo-electron microscopy (cryo-EM). The C-TEM of amoebae infected with the medusavirus showed four types of particles, i.e., pseudo-DNA-empty (p-Empty), DNA-empty (Empty), semi-DNA-full (s-Full), and DNA-full (Full). Time-dependent changes in the four types of particles and their intracellular localization suggested a new maturation process for the medusavirus. Viral capsids and viral DNAs are produced independently in the cytoplasm and nucleus, respectively, and only the empty particles located near the host nucleus can incorporate the viral DNA into the capsid. Therefore, all four types of particles were found outside the cells. The cryo-EM of these particles showed that the intact virus structure, covered with three different types of spikes, was preserved among all particle types, although with minor size-related differences. The internal membrane exhibited a structural array similar to that of the capsid, interacted closely with the capsid, and displayed open membrane structures in the Empty and p-Empty particles. The results suggest that these open structures in the internal membrane are used for an exchange of scaffold proteins and viral DNA during the maturation process. This new model of the maturation process of medusavirus provides insight into the structural and behavioral diversity of giant viruses.

**IMPORTANCE** Giant viruses exhibit diverse morphologies and maturation processes. In this study, medusavirus showed four types of particle morphologies, both inside and outside the infected cells, when propagated in amoeba culture. Time-course analysis and intracellular localization of the medusavirus in the infected cells suggested a new maturation process via the four types of particles. Like the previously reported pandoravirus, the viral DNA of medusavirus is replicated in the host’s nucleus. However, viral capsids are produced independently in the host cytoplasm, and only empty capsids near the nucleus can take up viral DNA. As a result, many immature particles were released from the host cell along with the mature particles. The capsid structure is well conserved among the four types of particles, except for the open membrane structures in the empty particles, suggesting that they are used to exchange scaffold proteins for viral DNAs. These findings indicate that medusavirus has a unique maturation process.

## INTRODUCTION

Medusavirus is a giant virus that was isolated from a hot spring water source in Japan (1) and subsequently propagated in *Acanthamoeba* culture, similar to other giant viruses reported to date (2–5). Because of its ability to convert host amoeba cells into a cyst, the virus was named after the mythical monster Medusa. Medusavirus has a genome of 381 kb, which encodes 461 putative proteins, 86 of which have their closest homologs in *Acanthamoeba*. Furthermore, the genome of its laboratory host, Acanthamoeba castellanii, encodes many medusavirus homologs, including the major capsid protein (MCP), suggesting that amoebae are the most promising natural hosts of medusavirus since ancient times, and lateral gene transfers have repeatedly occurred between the virus and amoebae (1). The genome of medusavirus encodes a complete set of histone proteins, including four core histones and one linker histone. Recently, a sister strain of medusavirus, named *Medusavirus stheno*, was isolated from a river near Kyoto, Japan, in which histone H3 and H4-encoding genes are fused together (6). Furthermore, the DNA polymerase of medusavirus is phylogenetically placed at the root of the eukaryotic clade. This suggests that medusavirus is closer to eukaryotes than to other viruses (1).

Medusavirus is taxonomically classified in the phylum *Nucleocytoviricota* (7), which is traditionally referred to as the nucleocytoplasmic large DNA virus (NCLDV), an expansive group of double-stranded DNA (dsDNA) viruses that possess a large particle size encapsulating a large genome (>100 kb) and infect various eukaryotic hosts (8, 9). The following families are classified in the phylum *Nucleocytoviricota*: *Ascoviridae*, *Asfarviridae*, *Iridoviridae*, *Marseilleviridae*, *Mimiviridae*, *Phycodnaviridae*, *and Poxviridae* (10). Medusavirus was recently classified in the phylum *Nucleocytoviricota* as an independent proposed family, *Medusaviridae* (1, 11).

Cryo-electron microscopy (cryo-EM) single-particle analysis (SPA) using a 200-kV electron microscope showed that the medusavirus virion is composed of an icosahedral shell with triangulation number (12) T of 277 (h = 7, k = 12) and exhibits a diameter of 260 nm between the opposing 5-fold vertices (1). The 8-nm single-layered major capsid is covered with 14-nm spherical-headed spikes extending from each capsomere. The viral capsid is backed by a 6-nm-thick internal membrane, which encloses viral DNA, as is commonly found in NCLDVs. The membrane extruded to the 5-fold vertices of the icosahedral capsid interacts directly with the capsid. The medusavirus particles purified in the laboratory are either filled with DNA or contain no DNA inside; however, the two types of particles are structurally similar, as shown by the ∼30-Å resolution cryo-EM maps.

Many NCLDVs form a local compartment within the host cytoplasm, called the viral factory, where the virus propagates efficiently (13). Medusavirus does not form such a viral factory in the host cytoplasm for virus replication (1). Fluorescent *in situ* hybridization (FISH) analysis of the medusavirus-infected cells revealed that the viral DNA first localized to the host nucleus. Then, the signals of the newly synthesized medusavirus DNA gradually became stronger at the periphery of the nucleus up to 8 h postinfection (hpi), indicating that the replication of the viral DNA starts in the host cell nucleus. At 14 hpi, the viral DNA signal spread to the cytoplasm of the host cells. The newly born virions were then released from host cells. Time-lapse phase-contrast light microscopy showed that the host amoeba cells exhibited intracellular bridge formation and rotational movement due to infection with the medusavirus, eventually causing the cells contract with death (14). This observation suggests that the nuclear function of amoeba cells is dominated by the replication of medusavirus and that progeny viruses cross the host cell membrane and are released at the same time. A recent study suggests that early expression of genes, including histone H1 of medusavirus, results in the remodeling of the host nuclear environment before medusavirus DNA replication (11). Together, these observations suggest that medusavirus exhibits unique characteristics with respect to particle maturation, compared with other NCLDVs. However, the morphological features of the medusavirus during maturation have not yet been investigated.

In the current study, we found four types of medusavirus particles (pseudo-DNA-empty [p-Empty], DNA-empty [Empty], semi-DNA-full [s-Full], and DNA-full [Full] particles) inside and outside the virus-infected amoeba cells. Time-course analysis of medusavirus-infected cells using conventional transmission electron microscopy (C-TEM) suggested that these four types of particles show the maturation process in the cell. Furthermore, the intracellular localizations of different types of medusavirus particles indicated that viral capsid and viral DNA are produced independently in the cytoplasm and nucleus, respectively, and only the empty particles located near the nucleus can incorporate viral DNA into the capsid. As a result, many immature particles, along with mature particles, are released from the host cell. Cryo-EM using a 300-kV electron microscope revealed that the medusavirus capsid was covered with three types of uniformly arranged spikes and the capsid structure was well conserved throughout the maturation process, except for a small difference in size, depending on the presence or absence of DNA. The internal membrane exhibited a structural array pattern similar to that of the capsid and included an open structure in the empty particles. The open membrane structure is presumably used for exchanging scaffold proteins and viral DNA during the maturation process. Based on these observations, we propose a new maturation model for medusavirus, which provides insight into the structural and behavioral diversity of giant viruses.

## RESULTS

### Formation of four types of medusavirus particles in the host cell during maturation.

**FIG 1:**
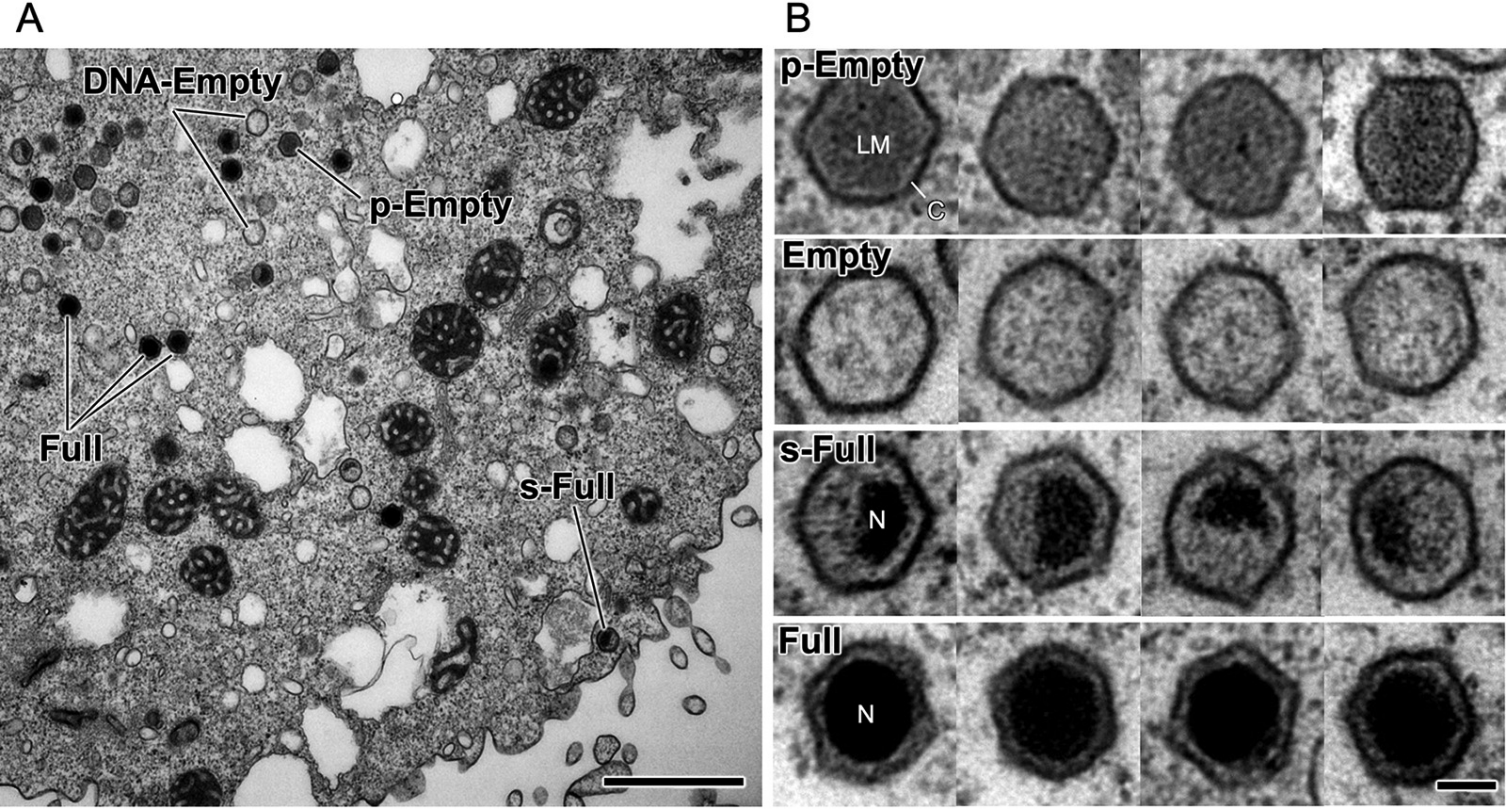
C-TEM image of amoeba cells infected with medusavirus. (A) A representative micrograph at 22 hpi. p-Empty, Empty, s-Full, and Full particles are labeled. (B) Zoom-in images of the four different types of medusavirus particles. p-Empty, capsid is filled with a low-density material; Empty, DNA-empty capsid; s-Full, partially filled with viral DNA; Full, capsid is completely filled with viral DNA. LM, low-density material; C, viral capsid; N, viral nucleoid. Scale bars = 1 μm (A) and 100 nm (B).

Amoeba cells infected with medusavirus were chemically fixed, embedded in plastic resin, and sliced into thin sections, which were then examined by C-TEM. Many replicated medusavirus particles were observed in the host cell cytoplasm at 22 hpi (Fig. 1A). The particles were classified into four different types, depending on their structure, i.e., p-Empty, Empty, s-Full, and Full (Fig. 1B). The p-Empty particles were filled with a low-density material (not viral DNA), whereas the Empty particles exhibited an empty capsid. The s-Full particles were partially filled with viral DNA, whereas the Full particles were completely filled with viral DNA. These particles were distributed in the cytoplasm at a certain rate, depending on the time point postinfection.

**FIG 2:**
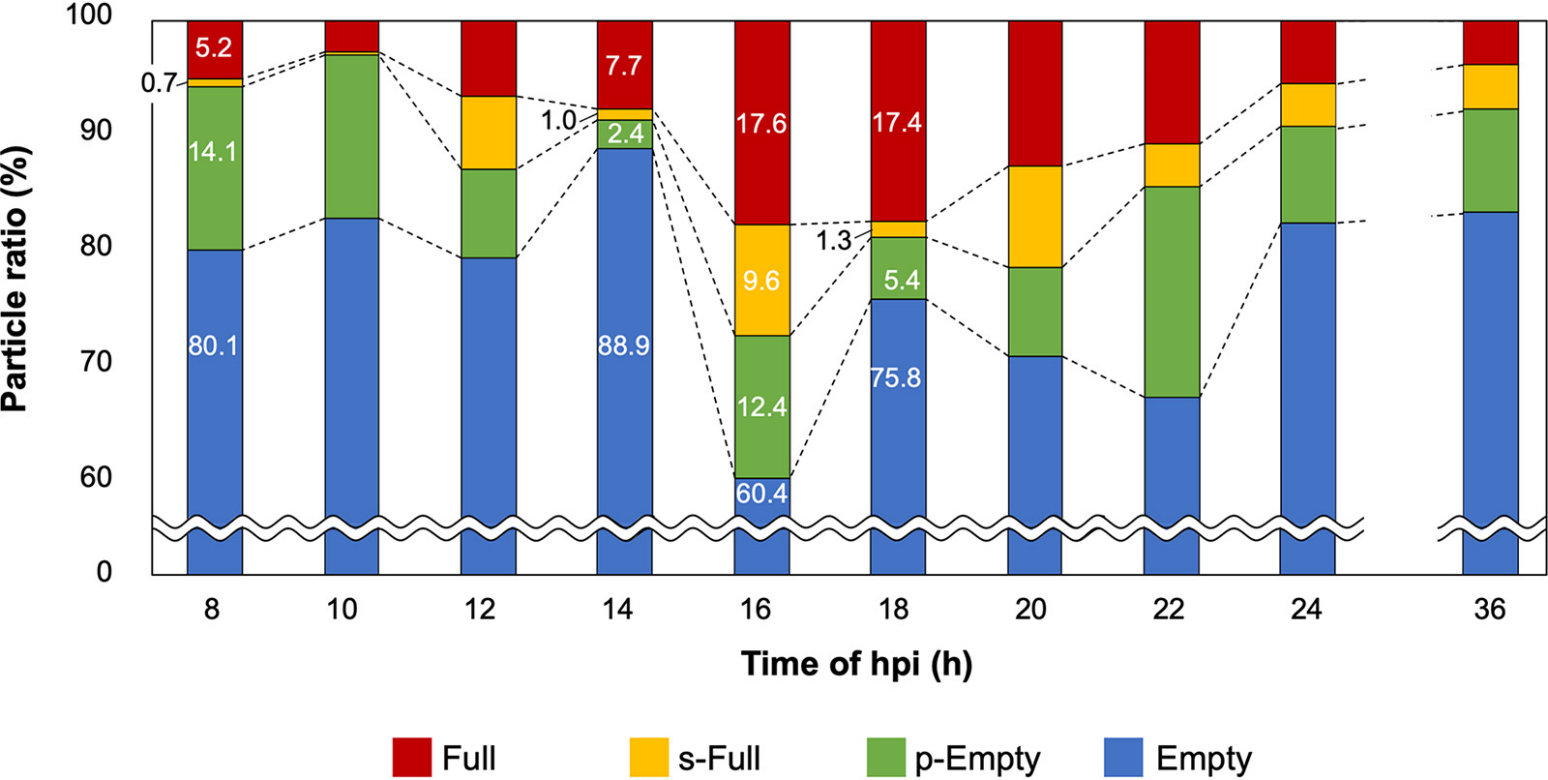
Time-dependent changes in the four types of replicated medusavirus particles in the infected host cells. Proportions of Full, s-Full, p-Empty, and Empty particles are indicated in red, yellow, green, and blue, respectively. The data before 8 hpi and from 24 to 36 hpi were omitted because the number of particles was insufficient or the morphological changes in the four types of medusavirus particles were not significant.

To determine the progression of one type of medusavirus particle to another during the maturation process, the medusavirus-infected amoeba cells were fixed every 2 h, and thin sections were observed by C-TEM. Figure 2 shows the change in the relative proportions of the four types of newly replicated medusavirus particles in the host cells over time. Data from 0 to 8 hpi were omitted because sufficient viral particles were not observed in the cytoplasm. The Empty and p-Empty particles (blue and green, respectively, in Fig. 2) accounted for more than 85% of all medusavirus particles up to 14 hpi; their proportions decreased significantly to less than 72% at 16 hpi and then increased again to more than 80% after 18 hpi. This oscillation suggests that the first major duplication and release of medusavirus virions occurred at 16 hpi. This result is consistent with a previous study, in which FISH analysis revealed that the newly synthesized viral DNAs are transferred to the cytoplasm at 14 hpi, and then virions appear extracellularly (1). The temporary increase in the number of Empty particles and decrease in the number of p-Empty particles at 14 hpi are probably the result of the conversion of p-Empty particles into Empty particles by the release of low-density material. The proportions of s-Full and Full particles (red and yellow, respectively, in Fig. 2) increased drastically at 16 hpi, whereas that of Empty particles decreased. This shows that the viral DNA is packaged in the empty capsid at this stage. Mature virions were released from host cells at 16 hpi. After 18 hpi, the proportions of all four types of medusavirus particles gradually reached a constant level, suggesting that the replication process of the late-infecting viruses overlaps that of the early-infecting viruses. Together, these observations suggest that the medusavirus maturation process proceeds as follows: (i) formation of p-Empty particles in the host cytoplasm, (ii) conversion of p-Empty particles into Empty particles by the release of low-density material from the capsid, (iii) uptake of viral DNA replicated in the host nucleus into the capsid of Empty particles, and (iv) production of Full particles via the s-Full stage. The temporary decrease in the proportion of p-Empty particles at 14 hpi, followed by a rapid recovery at 16 hpi, probably indicates a continuous production of procapsids (p-Empty) in the cytoplasm.

**FIG 3:**
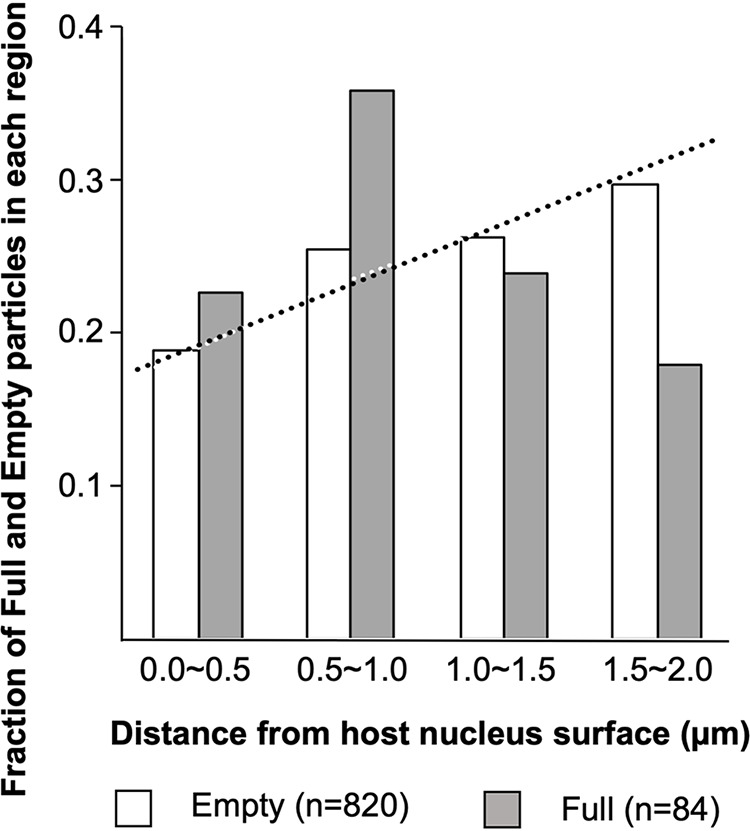
Intracellular distribution of Empty and Full medusavirus particles. The fractions of Empty and Full medusavirus particles in each region are shown relative to the distance from the host nucleus. The dotted line shows the distribution of medusavirus particles, assuming they are randomly distributed in the host cell cytoplasm, where the number of particles increases linearly with the increase in distance from the nuclear surface. The data were collected between 8 and 36 hpi.

Next, to determine where the empty medusavirus capsids incorporate viral DNA, we investigated the intracellular localization of Empty and Full particles. The distance of individual medusavirus particles from the host nuclear surface was measured, and the fractions of Empty and Full medusavirus particles in each region were plotted on a chart (Fig. 3). To avoid geometrical interference with the plasma membrane, only particles located at least 2 μm away from the plasma membrane are plotted on the chart. In addition, the observation area was minimized (up to 2 μm from the nucleus surface) to avoid interference from the cell surface, which may or may not appear in the upper and lower sections. Assuming that the viral particles were randomly distributed in the cytoplasm (random particle distribution), the number of particles increased linearly with the increase in distance from the nuclear surface (dotted line in Fig. 3). The increase in Empty particles was significantly consistent with the random particle distribution curve (white bars in Fig. 3), indicating that the Empty particles are randomly located in the cytoplasm. On the other hand, Full particles were predominantly located 0.5 to 1.0 μm from the nuclear surface, suggesting that Full particles (virions) were predominantly generated near the host nucleus surface by incorporating viral DNA in this region.

In these experiments, due to the use of thin sections of ∼70 nm, we cannot completely distinguish Empty, s-Full, and Full particles because only part of the particle was observed in these sections. To estimate how much error would occur if the 70-nm thin sections were used to count the four types of particles, a 300-nm-thick section of a medusavirus-infected amoeba cell sample was created; it included the entire medusavirus particles. Then, three-dimensional (3D) tomographic images were generated from the tilt-series images collected by 200-kV electron microscopes (see Movies S1 and S2 in the supplemental material). The four types of medusavirus particles were counted from the 3D tomograms. As a result, the numbers of the four types of particles in a total of 135 particles from the 18-hpi samples were as follows: 20 Full (14.8%), 3 s-Full (2.2%), 7 p-Empty (5.2%), and 105 Empty (77.8%) particles. These percentages were close to the data counted in the 70-nm thin sections of the 18-hpi samples, as shown in Fig. 2 (17.4% Full, 1.3% s-Full, 5.4% p-Empty, and 75.8% Empty particles). We concluded that the proportions of the four types of particles counted from the 70-nm thin sections do not cause serious problems in these experiments.

### Cryo-EM observations of medusavirus particles outside the host cells.

**FIG 4:**
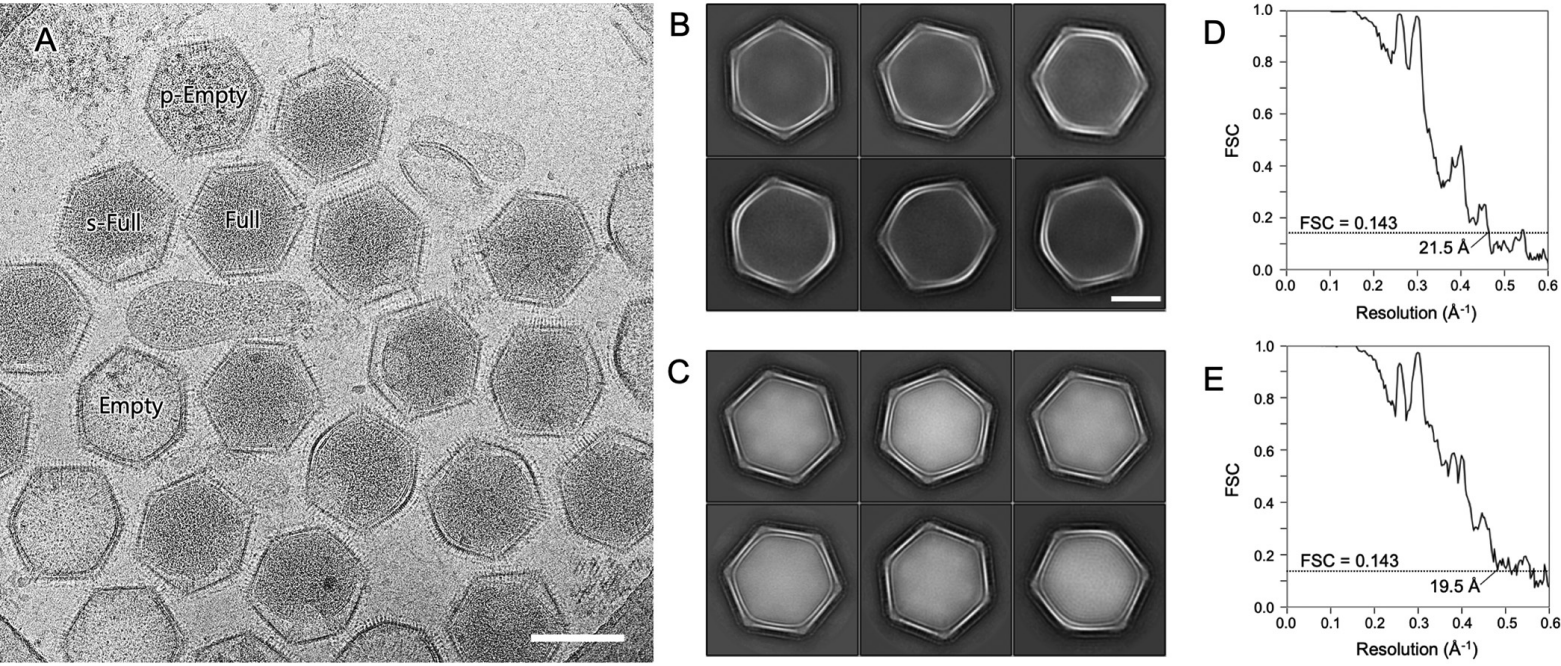
Cryo-EM SPA of the Empty and Full medusavirus particles using a 300-kV microscope. (A) A representative micrograph. Empty, p-Empty, Full, and s-Full particles are labeled. Scale bar = 200 nm. (B and C) Representative 2D classification images based on 4,551 Empty (B) and 6,981 Full (C) particles. Scale bar = 100 nm. (D and E) Gold standard FSC curves of the final 3D reconstruction maps of the Empty (D) and Full (E) particles. The resolution was estimated at 21.5 Å for Empty particles and 19.5 Å for Full particles using the 0.143 FSC criterion.

**FIG 5:**
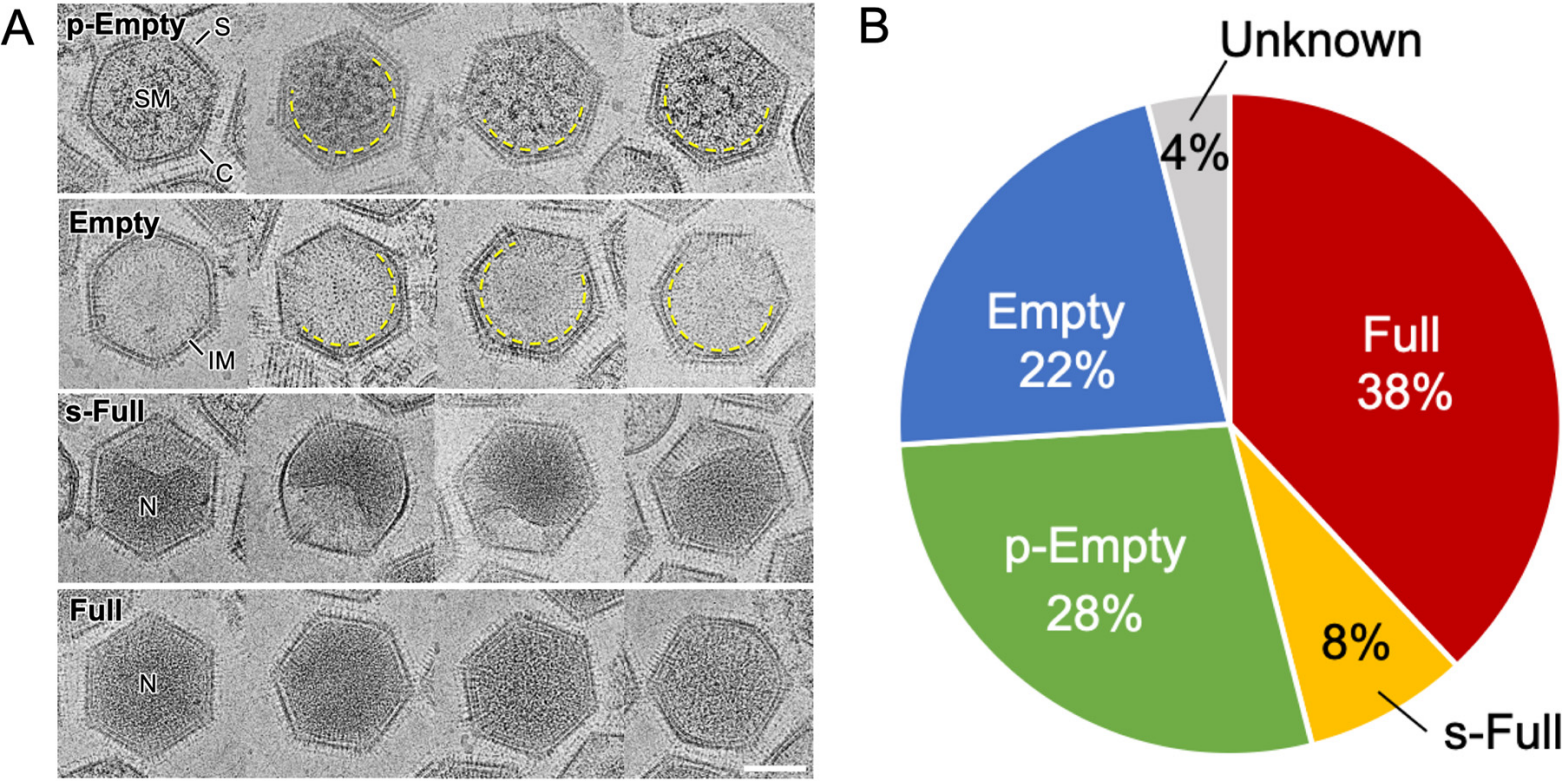
Four types of medusavirus particles observed outside the host cells. (A) Cryo-EM images of four different types of medusavirus particles, i.e., p-Empty, Empty, s-Full, and Full. The internal membrane (IM) showed a discontinuous structure in the s-Empty and Empty particles (dashed yellow curves). S, spike; C, capsid; SM, spongy material; N, nucleoid. Scale bar = 100 nm. (B) Fractions of the four types of medusavirus particles outside the host cells. The unknown category represents the unclassified broken particles.

Medusavirus particles were collected from the supernatant of medusavirus-infected amoeba cultures and observed by cryo-EM. The results clearly showed four types of medusavirus particles (Fig. 4A and Fig. 5A), similar to those identified inside the virus-infected host cells (Fig. 1B). Cryo-EM imaging, which did not involve chemical fixation and dehydration, revealed not only the fine capsid structure but also the morphological differences among the four types of medusavirus particles (Fig. 4A and 5A). The p-Empty particles were filled with a low-density (spongy) material that was not present in the Empty particles. Interestingly, the internal membrane of p-Empty and Empty particles was discontinuous (dashed yellow lines in Fig. 5A), whereas that of s-Full particles (with the partly incorporated DNA) was deformed (“N” in s-Full in Fig. 5A). The relative proportions of p-Empty, Empty, s-Full, and Full particles outside the host cells were 28%, 22%, 8%, and 38%, respectively (Fig. 5B); these fractions were significantly different from those inside the host cells (Fig. 2). The Empty and p-Empty particles (blue and green, respectively, in Fig. 5B) together represented 50% of all medusavirus particles outside the host cells, which was considerably lower than their proportion inside the host cells (>80%) except at 16 hpi. In contrast, Full and s-Full particles (red and yellow, respectively, in Fig. 5B) represented 46% of all medusavirus particles outside the host cells but were less than 20% of all medusavirus particles inside the host cells except at 16 hpi. The fraction of mature particles (virions) outside the host cells was greater than that inside the cells, suggesting that virions are selectively released from the cells.

### Visualization of open structures in the internal membrane by cryo-electron tomography.

**FIG 6:**
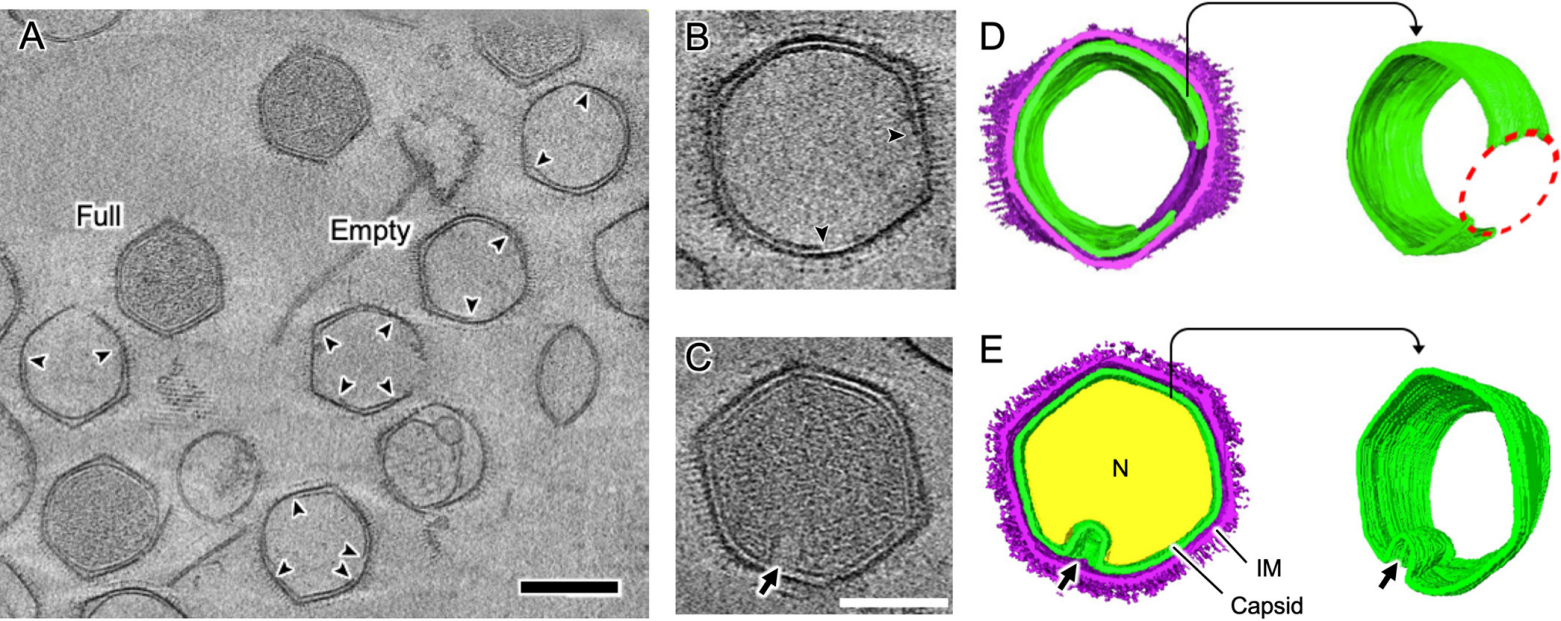
Structural analysis of the internal membrane of medusavirus particles by cryo-ET. (A) A tomogram slice of the different types of medusavirus particles outside the host cells. The internal membranes of the Empty particles are discontinuous (arrowheads). Representative Full and Empty medusavirus particles are labeled. Scale bar = 200 nm. (B and D) A tomogram slice (B) and its segmented volumes (D) of the Empty medusavirus particle. The discontinuous internal membrane shows an open structure in the membrane (red dotted circle). (C and E) A tomogram slice (C) and segmented volumes (E) of the s-Full medusavirus particle. The internal membrane is partially detached from the external capsid and deformed (arrow), but the membrane is completely closed. The capsid, internal membrane (IM), and nucleoid (N) are indicated in purple, green, and yellow, respectively. Scale bars = 200 nm (A) and 100 nm (C).

The discontinuous internal membrane (dashed yellow curves in Fig. 5A) is reminiscent of the formation of open structures in the Empty and p-Empty particles. Cryo-electron tomography (cryo-ET) was used to investigate the 3D structure of the discontinuous internal membranes in p-Empty and Empty medusavirus particles (Fig. 6; also see Movies S3 and S4). The internal membrane was discontinued at one or two places in Empty particles but was completely closed in Full particles (Fig. 6A to C). A representative Empty particle was selected and segmented in the tomogram (Fig. 6B and D). The results of cryo-ET showed that the Empty particle contained an open structure, whereas the external capsid was closed. It has been suggested that the open structure of the internal membrane in Empty particles is used for exchanging scaffold proteins and viral DNA, although how these molecules pass through the external capsid is not clear at this resolution. On the other hand, cryo-ET of a representative s-Full particle (Fig. 6C and E) showed that the internal membrane was partly detached from the external capsid and deformed, but it continuously surrounded the viral DNA. However, in s-Full particles, how the remaining viral DNA is acquired through the closed internal membrane remains unclear.

### Cryo-EM SPA of Empty and Full particles.

**FIG 7:**
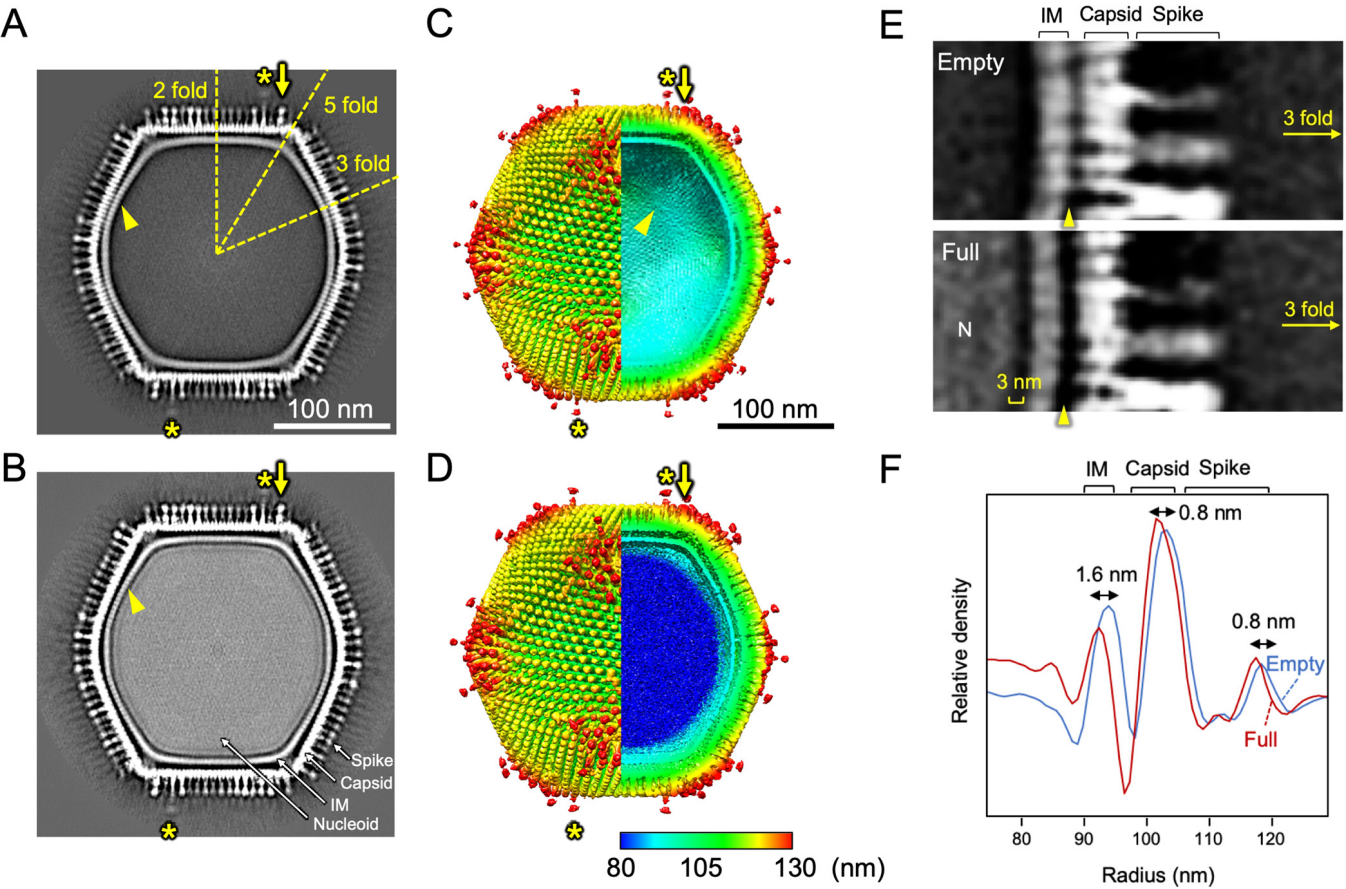
Cryo-EM 3D reconstructions of Empty and Full particles and their size comparison. (A to D) Center slices (A and B) and surface renderings (C and D) of the cryo-EM maps of the Empty (A and C) and Full (B and D) particles. The 3D maps were colored by radius. Asterisks indicate long spikes. Arrows indicate wide spikes. (E) Center slices of the Empty (top) and Full (bottom) particle cryo-EM maps near the 3-fold axis. Gaps between the capsid and internal membrane and between the internal membrane and nucleoid are indicated by the yellow bracket and yellow arrowhead, respectively. (F) Radial profile of the Empty (blue) and Full (red) particles. The size differences in the internal membrane (IM), capsid, and spikes are indicated.

The cryo-EM SPA of Empty and Full medusavirus particles was performed using a 300-kV microscope by imposing icosahedral symmetry. A total of 2,000 micrographs were analyzed (Fig. 4), and 4,551 Empty particles and 6,981 Full particles were manually selected for 3D reconstruction (Table 1). The two-dimensional (2D) class averages clearly showed whether the capsid of Empty and Full particles was empty or contained viral DNA (Fig. 4B and C). The resolution of these 3D reconstructed maps (Fig. 7A to D) was finally estimated at 21.5 Å for Empty particles and 19.5 Å for Full particles using the gold standard Fourier shell correlation (FSC) (Fig. 4D and E). As shown in the tomography map (Fig. 6), the medusavirus particles were relatively flexible, limiting the image resolution that could be achieved for both the Empty and Full particles. However, the cryo-EM maps provided detailed structural information for the medusavirus particles, as described below.

**TABLE 1:** Cryo-EM data set

Parameter	Finding
Microscope	Titan Krios G3
Accelerating voltage (kV)	300
Spherical aberration (mm)	0.1 (Cs corrector)
Detector	Falcon III
Total dose (e^−^/A^2^)	30
No. of micrographs	2,084
No. of frames per micrograph	40
Nominal magnification (×)	22,500
Pixel spacing on specimen (Å/pixel)	3.03
No. of initial particles picked	
Empty particles	4,625
Full particles	7,038
No. of final particles used	
Empty particles	4,551
Full particles	6,981
Symmetry imposed	
Empty particles	I1
Full particles	I1
Resolution (Å)	
Empty particles	21.5
Full particles	19.5

**FIG 8:**
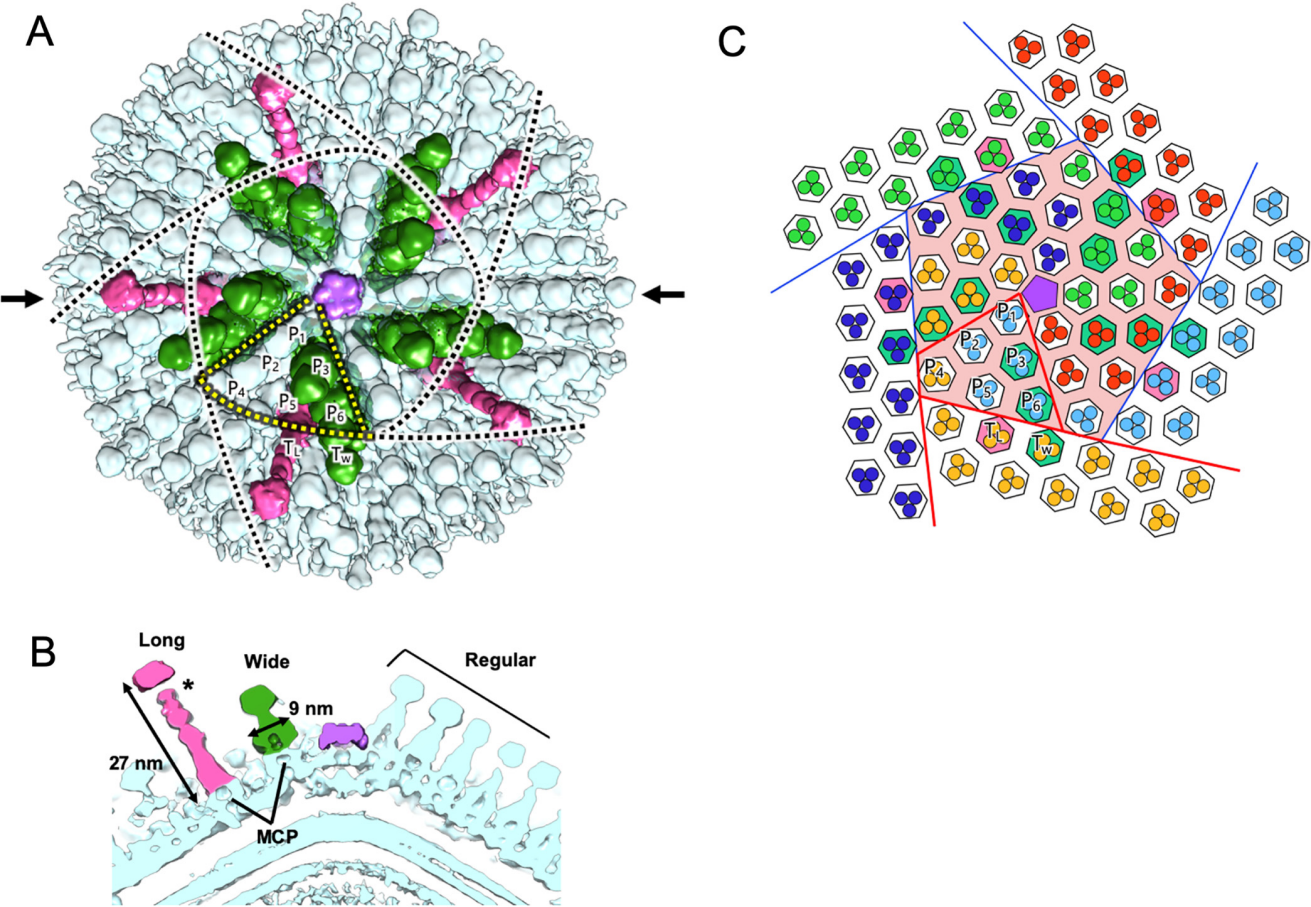
Three spike types around the 5-fold vertex. (A) A cryo-EM map isosurface viewed along the 5-fold axis. Long spikes (magenta), wide spikes (green), and regular spikes (cyan) are indicated. Penton is shown in purple. (B) Vertical slice of the cryo-EM map isosurface along the 5-fold axis cutoff at the arrows in panel A. The color scheme used for the three spike types and the penton is the same as in panel A. (C) Schematic drawing of panel A. The color scheme used to indicate the distribution of the three types of spikes on the hexagon is the same as in panels A and B. Orientations of MCP trimers are indicated using different colors. The central pentasymmetron (salmon pink) and surrounding five trisymmetrons are outlined using colored lines. Six capsomers (MCP trimer) in a triangular asymmetric unit (red-lined trapezoid) of the pentasymmetron are labeled P_1_ to P_6_. The long and wide spikes on a trisymmetron are labeled T_L_ and T_W_, respectively.

The first prominent structural feature was the presence of long or wide spikes that extended around the vertices of the capsid surface (asterisks and arrows in Fig. 7A to D), in addition to the regular spikes that covered the entire capsid. The long spikes were 27 nm in length (measured from the top of the MCP trimer) and 6 nm in width (same as the regular spikes) (magenta in Fig. 8A and B). On the other hand, the width of the wide spikes was 9 nm at the root, and their length was 13 nm (same as the regular spikes) (green in Fig. 8A and B). Interestingly, these special spikes were located separately between the pentasymmetron and trisymmetron (Fig. 8A and C). The triangular asymmetrical unit of the pentasymmetron consisted of six capsomers (MCP trimer), labeled P_1_ to P_6_, and two wide spikes located in P_3_ and P_6_ (Fig. 8A and C). The third wide spike (T_W_ in Fig. 8A and C) was located in the trisymmetron adjacent to the P_6_ wide spike, whereas the long spike (T_L_ in Fig. 8A and C) was located in the trisymmetron adjacent to the P_5_ regular spike. The wide spikes showed a wider root underneath the spherical head (green in Fig. 8A and B). The long spikes showed an extended tail underneath the spherical head, although the density was weak (asterisks in Fig. 7A and B and asterisk in Fig. 8B). The spherical heads of both long and wide spikes were wider than those of regular spikes (Fig. 8B). All types of spikes were found in both Empty and Full medusavirus particles (Fig. 7A to D), suggesting that spikes are formed at the early maturation stages. These spikes are possibly involved in host recognition and host membrane permeation, although further investigation is needed to verify their function.

**FIG 9:**
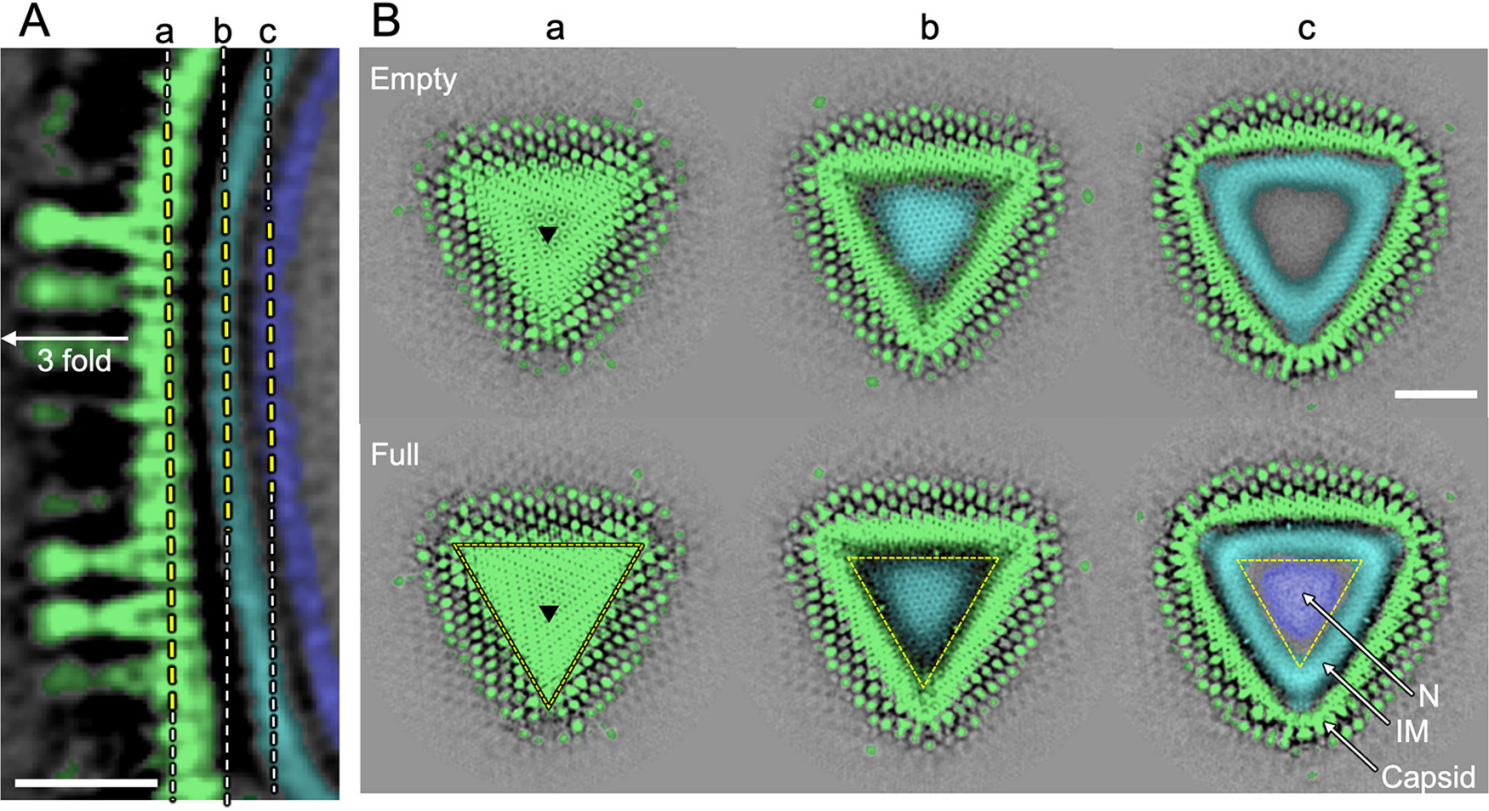
Surface slice views of medusavirus. (A) Center slice view of the medusavirus cryo-EM map near the 3-fold axis. The 3-fold axis is indicated. Scale bar = 20 nm. (B) Surface slice views of the Empty (top) and Full (bottom) particles at the dashed lines of a, b and c in panel A. Black triangles indicate the 3-fold axis. Scale bar = 50 nm. The capsid, internal membrane (IM), and nucleoid (N) are colored light green, cyan, and blue, respectively.

**FIG 10:**
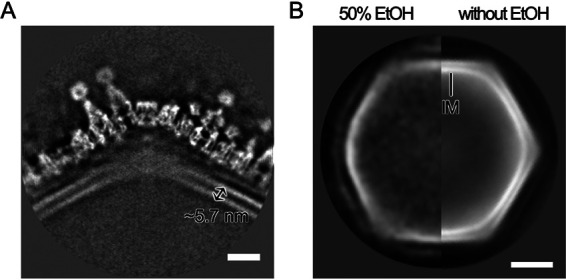
Structure and behavior of the internal membrane of medusavirus. (A) Central slice of the Empty medusavirus cryo-EM map at the 5-fold vertex, showing a typical lipid bilayer structure (width, ∼5.7 nm). Scale bar = 10 nm. (B) The 2D averaged image of Empty particles treated with and without 50% ethyl alcohol (EtOH). In the particles treated with 50% ethyl alcohol (left), the internal membrane (IM) disappeared. Scale bar = 50 nm.

**FIG 11:**
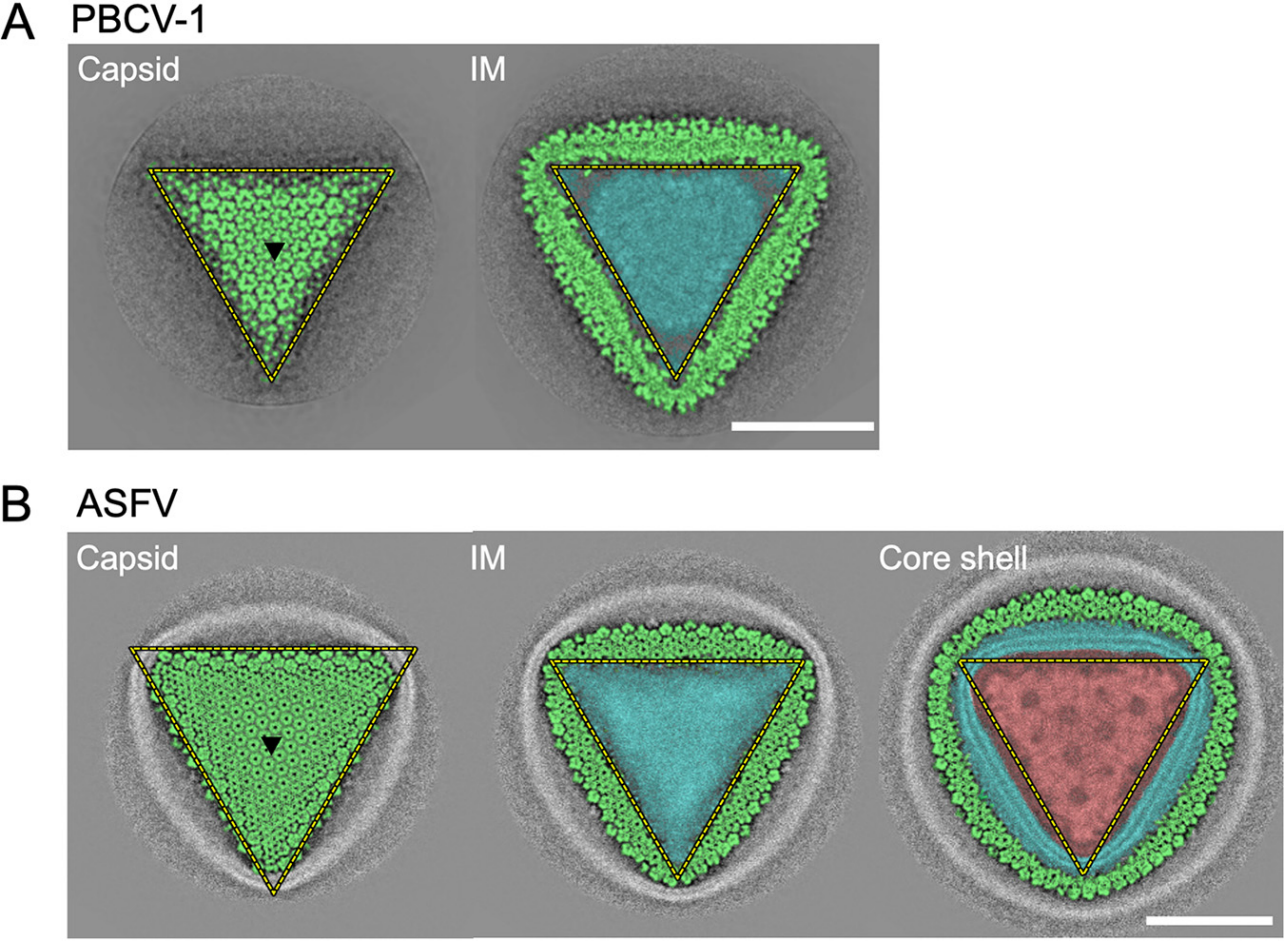
Surface slice views of PBCV-1 and ASFV at the 3-fold axis. (A) Surface slice views of PBCV-1 (EMD-5378 in the EMDB (https://www.ebi.ac.uk/emdb)) at the capsid and internal membrane layers. (B) Surface slice views of ASFV (EMD-0815) at the capsid, internal membrane, and core shell layers. The capsid, internal membrane (IM), and core shell are indicated in light green, cyan, and red, respectively. Black triangles indicate the 3-fold axis. Scale bars = 50 nm.

The second prominent structural feature was the lattice array on the internal membrane of the medusavirus particles (arrowheads in Fig. 7A and C), which showed similarity to the array of the external viral capsid (Fig. 9). The internal membrane had a width of 5.7 nm (Fig. 10A), which is typical of lipid bilayers, and looked similar to that of other NCLDVs (15–17). However, the lattice array on the internal membrane of medusavirus was unique and structurally reminiscent of the inner protein core shell of faustovirus (18) and African swine fever virus (ASFV) (Fig. 11B) (16, 17). To ensure that the internal membrane consists of a lipid membrane rather than a protein shell, medusavirus particles were treated with 50% ethyl alcohol. The 2D averaged images of the Empty medusavirus particles showed that the internal membrane disappeared following the alcohol treatment (Fig. 10B), suggesting that the internal membrane is composed of a lipid membrane rather than a protein shell. Based on these results, we concluded that the internal membrane of medusavirus consisted of a lipid bilayer containing a membrane protein, rather than only lipid, and exhibited a structural array pattern (Fig. 9Bb). The membrane proteins potentially allow the formation of open structures, as observed by cryo-ET, in the internal membrane (Fig. 6). Furthermore, a gap of approximately 3 nm was observed between the internal membrane and the nucleoid (yellow bracket in Fig. 7E), which has not been reported in other NCLDVs. This gap may be filled with low-density (spongy) materials, and the internal membrane indirectly encloses the viral nucleoid.

Unexpectedly, the Empty and Full medusavirus particles showed highly similar capsid structures but slightly different particle sizes (Fig. 7F). Compared with Empty particles, Full particles showed a 0.8-nm reduction in particle radius at the spike edges and a maximum radius reduction of up to 1.6 nm at the internal membrane region. The cryo-EM maps clearly showed that the gap between the internal membrane and the capsid was greater in Full particles than in Empty particles (arrowheads in Fig. 7E). This suggests that the strong interaction between the incorporated viral DNA and the internal membrane causes a virus particle contraction.

## DISCUSSION

In the present study, we first investigated the maturation process of medusavirus by performing a time course analysis of medusavirus-infected amoeba cells with C-TEM. We found four different types of particles inside the cells, namely, p-Empty, Empty, s-Full, and Full particles, and these time dependency and cytoplasmic localization analyses suggested that the medusavirus maturation process takes place in this order inside the host cell. These particles were also found in the supernatant of the amoeba culture, and the fine structures of Empty and Full particles were examined and compared by cryo-EM SPA, showing that the capsid structure is well preserved during the maturation process, except for a small change in size. However, the open membrane structures observed in Empty and p-Empty particles were suggested to be used for exchanging scaffold protein and viral DNA. Overall, this study reveals a new particle formation and maturation process of medusavirus.

In cryo-EM, the capsid of the p-Empty medusavirus particles was filled with a low density and spongy material rather than viral DNA. The actual function of p-Empty particles is not clear at this moment, but we can infer its biological role in the medusavirus particle maturation from some evidence. The low density and spongy material in p-Empty particles is reminiscent of the scaffold protein initially reported in dsDNA phages during capsid assembly (19). These phages use scaffold proteins to build a uniform icosahedral capsid and then release the proteins after the assembly of the capsid is complete and before the viral DNA is incorporated into the capsid. In NCLDVs such as mimivirus (20), ASFV (21), vaccinia virus (22), and Paramecium bursaria chlorella virus (PBCV-1) (23), the formation of virus particles begins with a membrane sheet scaffolded with proteins, and the resultant curled membrane sheet is used as a template for capsid assembly. Viral DNAs are then incorporated into the capsid by replacing the scaffold protein before the capsid is completely closed. All of these processes occur at the periphery of the viral factory. In the case of medusavirus, which does not form a viral factory, such a capsid formation process occurs in the host cytoplasm. The time course analysis of the medusavirus particles in the host cell (Fig. 2) showed that p-Empty particles dramatically decrease from 14.1% to 2.4% in the early stage of the replication cycle, between 8 and 14 hpi. In contrast, the Full particles increase from 5.2% to 17.6% during this period of 8 to 16 hpi. These findings suggest that the p-Empty particles are transformed into Full particles via Empty and s-Full particles at this stage. The fact that the cytoplasm is always filled with 60% to 80% Empty particles possibly indicates that the scaffold protein in p-Empty particles is released rapidly after building the capsid. The open structures of the internal membrane in the Empty and p-Empty particles (Fig. 5 and 6) may allow the release of this scaffold protein and subsequent uptake of viral DNA, although the underlying mechanism remains unclear.

Careful observation of the open membrane structures in the medusavirus capsid revealed that the membranes are always terminated near the outer capsid (Fig. 5A and 6). The cryo-EM map of medusavirus showed an array pattern on the internal membrane similar to that on the outer capsid (Fig. 7 and 9), suggesting that membrane proteins contained in the internal membrane strongly interact with the outer capsid. PBCV-1 and ASFV, whose structures were reported at a higher resolution (15, 16), exhibited a smooth internal membrane (Fig. 11) and did not show an open membrane structure. Membrane proteins in the internal membrane probably control the open structures by collaboratively interacting with the outer capsid.

**FIG 12:**
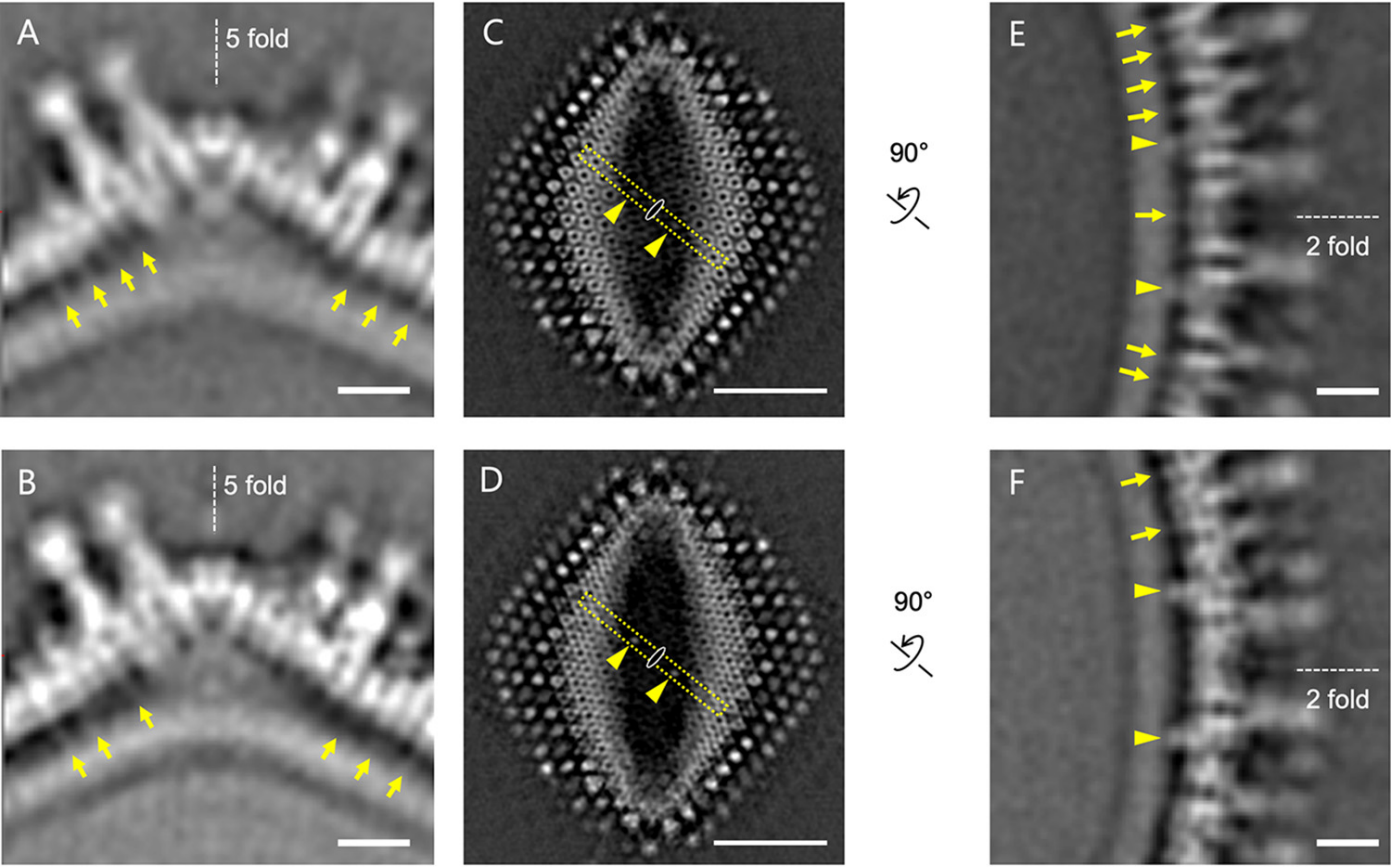
Interconnecting components between the capsid and internal membrane in medusavirus. (A and B) Center slices of the Empty (A) and Full (B) particles at the 5-fold vertices. Arrows indicate the thread-like interconnecting structures between the extruded internal membrane and the capsid. The 5-fold axis is indicated. Scale bars = 10 nm. (C and D) Surface slices of the Empty (C) and Full (D) particles at the 2-fold axis. Arrowheads show two symmetry-related (yellow dotted box) interconnecting structures. The 2-fold axis is indicated. Scale bars = 50 nm. (E and F) Center slices of the Empty (E) and Full (F) particles at the 2-fold axis, which are rotated 90° from the yellow-boxed areas of panels C and D, respectively. The interconnecting thread-like structures and symmetry-related interconnecting structures are indicated by arrows and arrowheads, respectively. The 2-fold axis is indicated. Scale bars = 10 nm.

The cryo-EM reconstructions of the medusavirus at ∼20-Å resolution revealed no significant structural differences between the Empty and Full particles other than the presence or absence of the viral DNA, but the sizes of the two particles showed a slight difference (Fig. 7E and F). Interestingly, the radius of the internal membrane of the Full particles is 1.6 nm shorter than that of the Empty particles, while the radii of the capsid and the outermost spikes of the Full particles are 0.8 nm shorter than those of the Empty particles. This finding suggests that the internal membrane strongly interacts with the nucleoid and, in the presence of the nucleoid, causes a major contraction in the internal membrane region. However, the space between the internal membrane and the nucleoid is 3 nm (yellow bracket in Fig. 7E), and no interconnecting components are observed. On the other hand, a minor contraction of the radii of the capsid and the outermost spikes (by 0.8 nm) also suggests an interaction between the internal membrane and the outer capsid. The cryo-EM maps showed several interconnecting components between these two structures (Fig. 12). At the 5-fold vertices, several thread-like structures were observed between the extruded internal membrane and the capsid in both Empty and Full particles (arrows in Fig. 12A and B). Around the 2-fold axes of the particle surface, in addition to the thread-like structures (arrows in Fig. 12E and F), two symmetrically related interconnecting structures are clearly identified in both Empty and Full particles (arrowheads in Fig. 12C to F). These interconnecting components yield relatively weak interactions between the internal membrane and the external capsid.

Cryo-EM using a 300-kV microscope revealed for the first time long and wide spikes of the medusavirus particle, in addition to the normal spikes. The asymmetric units around the 5-fold vertices of the capsid include three wide spikes (P_3_, P_6_, and T_W_ colored green in Fig. 8A), i.e., two on the asymmetric unit of the pentasymmetron (P_3_ and P_6_ in the yellow dotted area in Fig. 8A) and one on the adjacent trisymmetron (T_W_ in one black dotted area in Fig. 8A). On the other hand, one long spike (T_L_ colored magenta in Fig. 8A) in the asymmetric unit was located adjacent to the wide spike on the same trisymmetron (T_W_ in Fig. 8A and C). These different types of spikes were located adjacent to each other, but no interaction was found between these spikes. Xiao and coworkers reported that, in *Cafeteria roenbergensis* virus and PBCV-1, the orientation of the MCP trimers is constant in each trisymmetron and in each asymmetric unit of the pentasymmetron, except for the MCP trimers located at five corners of the pentasymmetron (P_4_ in Fig. 8C), and these MCP trimer arrays are interconnected in 60° orientation, eventually forming a large protein cage (24). This characteristic MCP trimer array has also been confirmed in ASFV (16) and marseillevirus (25), and our results showed that medusavirus also employs this scheme of capsomere arrays (Fig. 8C). The resultant MCP trimer arrangement suggests that the wide spikes in the asymmetric unit of the pentasymmetron (P_3_ and P_6_ in Fig. 8C) are oriented 60° from the wide and long spikes in the adjacent trisymmetron (T_W_ and T_L_ in Fig. 8C). The function of these special spikes is currently unknown, and further investigation is needed to understand the biological significance of the spike orientation and function.

The intracellular localization of Full particles was predominantly near the host nucleus (Fig. 3), suggesting that the DNA packaging of medusavirus occurs in the region surrounding the host nucleus. Medusavirus has not been reported to form a viral factory in amoebae (1). These results suggest that the viral capsid and DNA are independently produced in the host cytoplasm and nucleus, respectively, and that only empty particles located near the host nucleus can take up the viral DNA. As a result, DNA packaging is relatively inefficient with medusavirus, compared to other NCLDVs that form a viral factory (13). In iridovirus (26) and PBCV-1 (23), which form a viral factory near the host nucleus, capsid production occurs in the viral factory, and viral DNA replicated inside the host nucleus is delivered to the viral factory for encapsulation by the capsid. Mimivirus (20) and vaccinia virus (27), which also form a viral factory, produce the capsids around the viral factory and incorporate the viral DNA that is replicated in the viral factory itself. Thus, these NCLDVs package DNA with greater efficiency than medusavirus, in which DNA packaging accidentally occurs near the host nucleus because of the random distribution of empty particles in the cytoplasm (Fig. 3). On the other hand, pandoravirus does not create a viral factory in the host cytoplasm (4, 28). Pandoravirus enters the host ameba cell and releases the viral DNA into the host cytoplasm. The DNA is delivered to the host nucleus, where a copy of the viral DNA is replicated. After that, the host nucleus disappears and new virions appear around the area previously occupied by the nucleus, where the peripheral tegument formation and the viral DNA encapsulation occur simultaneously. Medusavirus exhibits similar DNA replication in the host nucleus, but capsids are formed independently in the cytoplasm and uptake of the viral DNA occurs outside the host nucleus. Therefore, medusavirus releases many immature particles of p-Empty, Empty, and s-Full particles from the host cells in addition to mature Full particles (Fig. 4 and 5). However, the proportion of Full particles outside the host cells (38%) is significantly greater than that in the host cell cytoplasm (<17.6%) (Fig. 2 and 5B). Medusavirus may have a mechanism for selectively releasing mature virions. Overall, these results indicate that medusavirus employs a unique strategy for maturation.

**FIG 13:**
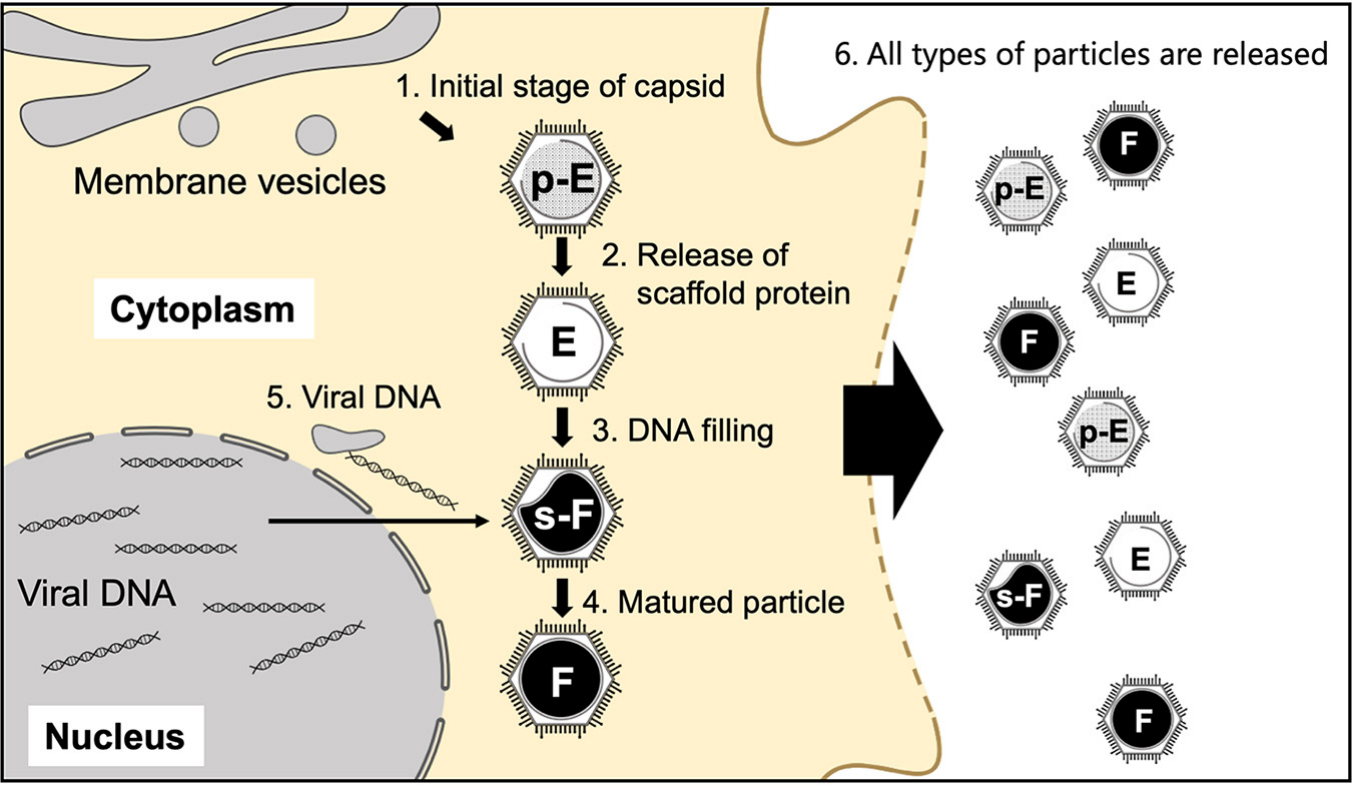
Proposed maturation process of medusavirus. Step 1, p-Empty (p-E) particles are initially generated in the cytoplasm. Step 2, p-Empty particles transform to Empty (E) particles by releasing spongy material (scaffold protein). Step 3, Empty particles incorporate viral DNA near the host nucleus (s-Full [s-F] and Full particles). Step 4, Full (F) particles are generated. Step 5, the open structures in the internal membrane can be closed with an extra membrane brought into the capsid along with the viral DNA. Step 6, mature virions are released from the host cells together with the immature p-Empty, Empty and s-Full particles.

Based on our observations of medusavirus, we propose a new maturation process for giant viruses (Fig. 13), as follows. (i) p-Empty particles filled with a low density and spongy material are initially produced in the cytoplasm by partially incorporating the host intracellular membrane. (ii) The p-Empty particles then turn into Empty particles by releasing the spongy material (scaffold protein). (iii) The Empty particles located near the host nucleus incorporate viral DNA independently generated in the host nucleus and transform into s-Full particles. (iv) The s-Full particles completely encapsulate the viral DNA to form Full particles. The open structures of the internal membrane are likely used to exchange the scaffold proteins with the viral DNA, but the underlying mechanism remains unknown. (v) The open membrane structures can be closed with an extra membrane brought into the capsid along with the viral DNA. (vi) Finally, the mature Full particles, together with immature p-Empty, Empty, and s-Full particles, are released from the amoeba cell. The cryo-EM SPA of medusavirus particles outside the host cells showed that all four types of particles possess a perfect set of spikes on the capsid. The spikes in progeny viruses are generally inactive in the host cell to avoid reaction with the host and must be activated before being released from host cells, as reported for dengue and Zika viruses (29, 30). Small structural modifications in the spikes may occur at the molecular level, or the spike structures inside the host cells may be different from those outside the host cells; however, these structural modifications cannot be identified at the current resolution and with the current methodology. Increases in the proportions of Full and s-Full particles outside the host cells may be a result of the structural modification in the spikes, which triggers the selective release of mature particles. To clarify the mechanisms underlying the medusavirus maturation process, it is necessary to separately investigate four types of medusavirus particles. We tried to separate four types of particles using sucrose density gradient centrifugation, but it did not work. Further techniques need to be adopted for this study in the future.

**FIG 14:**
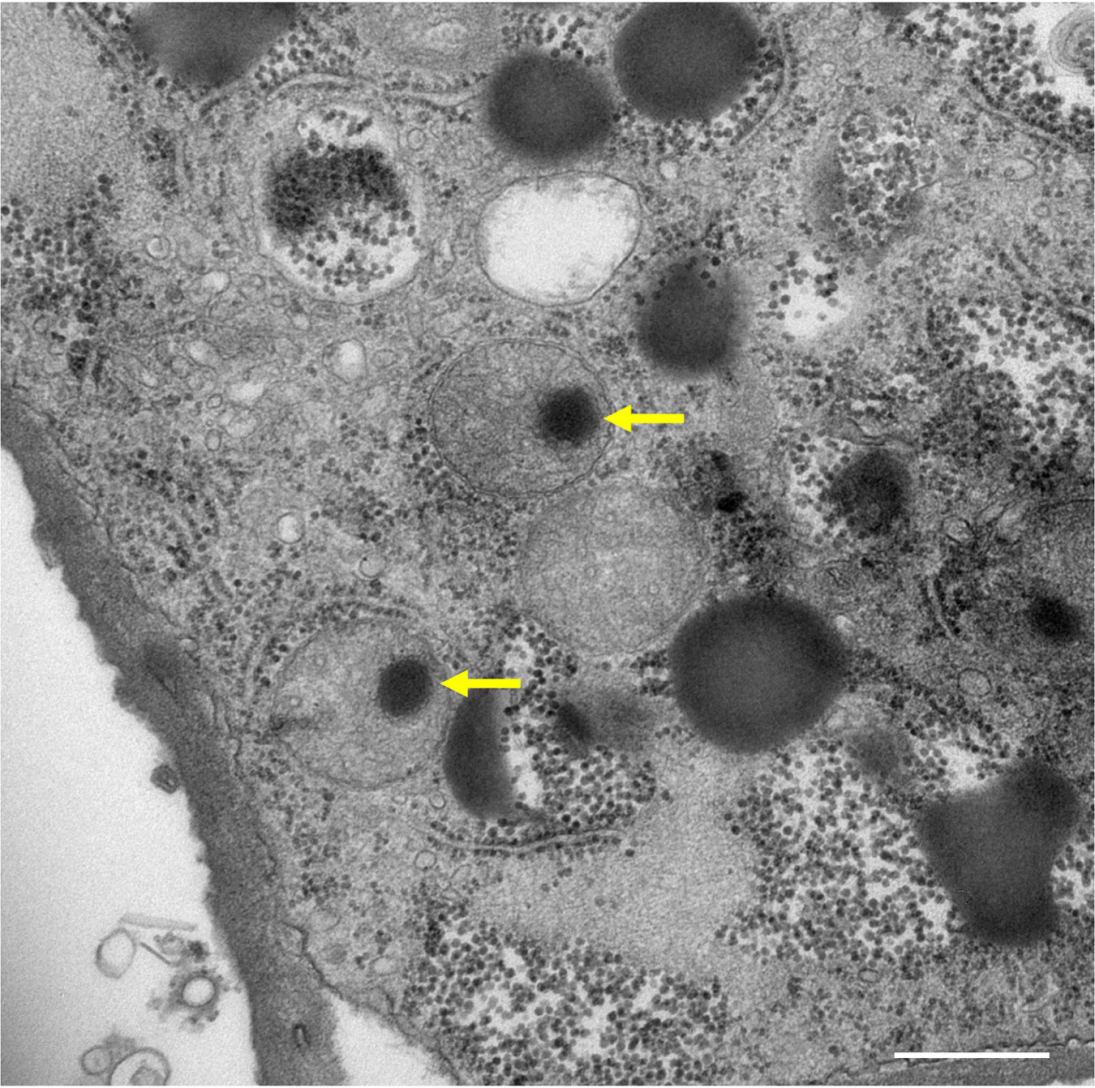
C-TEM image of a cyst amoeba induced by medusavirus infection. Medusavirus-like densities are often observed in mitochondria (yellow arrows). Scale bar = 500 nm.

During the preparation of this report, we heard a new report that clandestinovirus is phylogenetically most closely related to medusavirus and was considered a member of the proposed *Medusaviridae* family (31). Like medusavirus, clandestinovirus possesses a genome encoding four core histones and one homolog of mitochondrial chaperon BCS1. Further, clandestinovirus has a total of 10 proteins that function in mitochondria, suggesting that the virus regulates the host cell cycle and mitochondrial activity. Medusavirus is also known for its ability to convert host amoeba cells into cysts (1). In the cyst amoebae, infected medusaviruses are often found in mitochondria (Fig. 14; also see Movies S5 and S6 in the supplemental material), suggesting that medusavirus regulates mitochondrial activity by directly getting into the host mitochondria. Clandestinovirus, on the other hand, exhibits many morphologies and behaviors different from those of medusavirus. The genome of clandestinovirus is 65% larger than that of medusavirus, but the virion shows a much smaller particle size of 180 nm. In addition, clandestinovirus is replicated in a viral factory in *Vermamoeba vermiformis*, while medusavirus is replicated in Acanthamoeba castellanii without a viral factory. These viruses, which originated from a common origin, might have evolved over time in completely different environments.

In our first report, the Full and Empty medusavirus particles were described at 31.3-Å and 31.7-Å resolution, respectively, by cryo-EM SPA using a 200-kV microscope (1). The cryo-EM maps determined the triangulation number of the medusavirus icosahedral capsid as 277 and revealed the spiky feature of the capsid and the characteristic extrusion of the internal membrane structure into the 5-fold vertices of the capsid. In the current study, the resolution was improved to 19.5 Å and 21.5 Å for Full and Empty particles, respectively, by using a 300-kV microscope. The new cryo-EM maps revealed the presence of long and wide spikes in addition to the regular spikes and a membrane protein array in the internal membrane and its variable interactions with the outer capsid. Subnanometer structures of the icosahedral giant viruses have been reported in recent years, including a 3.5-Å structure of PBCV-1 (15), 4.6-Å and 4.8-Å structures of ASFV (16, 17), a 4.4-Å structure of melbournevirus (25), a 7.7-Å structure of tokyovirus (32), and a 8.6-Å structure of Singapore grouper iridovirus (33). These cryo-EM single-particle reconstructions were generated using ≥300-kV microscopes to avoid the defocus gradient and to keep enough electron transparency for large objects (34). Furthermore, some image-processing techniques, such as Ewald sphere correction (35), magnification anisotropy, and higher-order aberration correction (36), were applied to improve the resolution. For structures with resolution exceeding 5 Å, a method of block-based reconstruction was also adopted (37). In those cryo-EM SPAs, however, the initial map resolution reached approximately 1 nm without image correction techniques. In contrast, the initial resolution of the cryo-EM maps of medusavirus is much lower than 1 nm, primarily due to the capsid distortion. Compared with giant viruses with higher-resolution structures, the capsid structures of medusavirus with spikes are relatively flexible, degrading the achievable resolution. To obtain flexible domain structures of proteins, an image-processing technique of focused classification and refinement has been developed and tested (38). This technique may enhance the image resolution of medusavirus in the future.

## MATERIALS AND METHODS

### Growth and purification of medusavirus.

A. castellanii strain Neff was used as the viral host. Amoeba cells were cultured at 26°C in flasks containing 100 mL of peptone-yeast-glucose (PYG) medium, as described previously (1). A total of four cultures were grown and inoculated with medusavirus at a multiplicity of infection (MOI) of 1 to 2. The newly born medusaviruses were harvested 3 days postinfection. Amoeba cells and cell debris were removed by centrifugation (800 × *g* for 5 min at 24°C), and the medusavirus particles were concentrated by an additional centrifugation (8,000 × *g* for 35 min at 4°C). The viral particles were suspended in 10 mL phosphate-buffered saline (PBS) and filtered using a 0.45-μm filter (Millex-AA; Merck Millipore, Darmstadt, Germany). The filtered viral particles were centrifuged (8,500 × *g* for 35 min at 4°C) and resuspended in 10 μL of PBS. This process was repeated 5 to 10 times to obtain a sufficient amount of medusavirus particles.

### Time-course analysis of infected amoeba cells by C-TEM.

Medusavirus-infected amoeba cells were harvested at 2-h intervals and fixed with 2% glutaraldehyde in PBS at room temperature (23°C) for 30 min. The cells were washed three times with PBS and then postfixed with 2% osmium tetroxide in PBS at room temperature (23°C) for 1 h. The fixed cells were dehydrated using an ethanol gradient (50%, 70%, 80%, 90%, 95%, and 100% for 5 min each) at room temperature (23°C). The dehydrated cells were infiltrated with propylene oxide and embedded in epoxy resin mixture (Quetol 812; Nissin EM Co. Ltd., Tokyo, Japan). The resin was polymerized at 60°C for 2 days. Ultrathin sections (approximately 70-nm thickness) were prepared with a diamond knife using an ultramicrotome (EM-UC7; Leica Microsystems, Austria). The sections were stained with 2% uranyl acetate for 5 min and then with 0.4% lead citrate for 1 min. The stained sections were visualized using a JEM1010 microscope (JEOL, Ltd., Tokyo, Japan) at an acceleration voltage of 80 kV.

### Cryo-EM and SPA.

A 2.5-mL suspension of the purified medusavirus particles was applied to a Quantifoil grid (R1.2/1.3 Mo; Quantifoil Micro Tools GmbH, Germany), which was glow-discharged beforehand for 30 s. The grid was then blotted with filter paper (blotting time, 7 s; blotting force, 10 in the Vitrobot setting) and plunge-frozen at 4°C under 95% humidity using a Vitrobot Mark IV (Thermo Fisher Scientific, USA). The frozen grid was imaged using a Titan Krios G3 microscope at an acceleration voltage of 300 kV (Thermo Fisher Scientific). Movies were recorded on a Falcon III detector at a nominal magnification of ×22,500, corresponding to 3.03 Å per pixel on the specimen. A low-dose method (exposure at 10 electrons per Å^2^ per second) was used, and the total number of electrons accumulated on the specimen was ∼30 electrons per Å^2^ for a 3-s exposure time. A GIF Quantum energy filter (Gatan, Inc., USA) was used with a slit width of 20 eV to remove inelastically scattered electrons. Individual micrograph movies were subjected to per-frame drift correction by MotionCor2 (39), and the contrast transfer function parameters were estimated by CTFFIND4 (40).

To perform SPA, 4,625 Empty particles and 7,038 Full particles were manually selected and extracted from 2,084 motion-corrected images using RELION3.0 software (41). Subsequently, 4,551 Empty particles and 6,981 Full particles were selected from the extracted particles by 2D classification (Fig. 4A to C) and used for 3D reconstruction imposing icosahedral symmetry. The resolution of the final 3D maps was estimated at 21.5 Å for Empty particles and 19.5 Å for Full particles using the 0.143 gold standard FSC criterion (42) (Fig. 4D and E). The 3D reconstructions were visualized using UCSF Chimera software (43).

### Radial profile of Full and Empty particles.

The 5-fold axis of Full and Empty particles was reoriented to the front, and a center slice of the *z* axis was extracted from the reoriented volumes. The sliced image was rotationally averaged using EMAN2 software (44). Then, the radial profile was calculated using Fiji image analysis software suite (45).

### Cryo-ET.

Colloidal gold particles (15 nm) were mixed with the sample as a fiducial marker before freezing. The frozen grid was examined with a CRYO ARM 300 electron microscope (JEOL, Ltd.) at 300-kV accelerating voltage. Tilt-series images were collected in the range from −60° to +60° with a 2° increment using a low-dose mode, in which the total electron dose for 61 images was less than 100 e^−^/Å^2^ on the specimen. Images were recorded with a K3 camera (Gatan, Inc.) at a nominal magnification of ×15,000 and a pixel size of 3.257 Å on the specimen using the batch tomography procedure of Serial EM software (46). Image alignment and tomographic reconstruction were performed with IMOD software version 4.7.15 (47) using fiducial markers. The final tomograms were calculated with the simultaneous iterative reconstruction technique (SIRT) using images of 6.51 Å per pixel after application of a pixel binning of 2. The image segmentation in the 3D reconstructions was performed with Amira version 5.4.5 (Thermo Fisher Scientific).

### Electron tomography of a thick plastic section.

Thick sections (thickness, 300 nm) were obtained using a diamond knife and placed onto a single-slot grid. The grids were stained with 2% uranyl acetate for 5 min and then with 0.4% lead citrate for 1 min. The poststained specimen grid was observed with a JEM2200FS electron microscope (JEOL, Ltd.) equipped with a field emission electron source operating at 200 kV and an omega-type in-column energy filter (slit width, 20 eV). The images were recorded on a DE-20 direct detector camera (Direct Electron, San Diego, CA, USA) at a nominal magnification of ×20,000, resulting in a pixel spacing of 2.82 Å. For electron tomography, tilt-series images were collected manually in a range of approximately ±60° at 2° increments. The tilt-series images were aligned without fiducial markers, and tomograms were reconstructed using SIRT in IMOD software (47) with a pixel binning of 5.

### Electron tomography of cyst amoebae generated by cryofixation and freeze-substitution.

To clearly observe medusavirus-like particles in the mitochondria of cyst amoeba cells, cryofixation and freeze-substitution were applied. Amoeba cells infected with medusavirus showing cyst formation were frozen with an EM PACT high-pressure freezer (Leica Microsystems, Vienna, Austria). The frozen cells were transferred to cold acetone (–80°C) containing 1% osmium tetroxide and kept for 72 h for cryofixation. The temperature was then manually increased in a stepwise manner (–20°C for 1 h, 4 to 10°C for 0.5 h, and 22°C for 0.5 h). The cells were washed with 100% acetone at room temperature (22°C) and embedded in an epoxy resin mixture (Quetol 812; Nissin EM Co. Ltd.). The resin was polymerized at 60°C for 2 days. Thin sections (thickness, 200 nm) were obtained, and tomography was performed in the same way as described in the previous section.

### Data availability.

The density maps have been deposited in the EMDB (https://www.ebi.ac.uk/emdb) with the accession codes EMD-32073 (Full particle), EMD-32072 (Empty particle), and EMD-32071 (tomography).
