# Supplementary material for: Particle Morphology of Medusavirus Inside and Outside the Cells Reveals a New Maturation Process of Giant Viruses
Source: J Virol. 2022 Mar 17;96(7):e01853-21. doi: 10.1128/jvi.01853-21 (PMC9006890; doi:10.1128/jvi.01853-21)
Supplement: Supplemental file 1 — Supplemental Movie Legends. Download jvi.01853-21-s0001.pdf, PDF file, 0.04 MB [file jvi.01853-21-s0001.pdf]

Supplementary information

Movie S1: A tilt series of medusavirus particles inside the host cell.

Movie S2: A tomogram slices of medusavirus particles inside the host cell.

Movie S3: A tilt series of medusavirus particles located outside the host cell.

Movie S4: A tomogram slices of medusavirus particles located outside the host cell.

Movie S5: A tilt series of cyst amoeba generated by cryo-fixation and freeze substitution. A medusavirus-like density is observed in mitochondria.

Movie S6: A tomogram slices of cyst amoeba generated by cryo-fixation and freeze substitution. A medusavirus-like density is observed in mitochondria.
